# Proteomics study of changes in soybean lines resistant and sensitive to *Phytophthora sojae*

**DOI:** 10.1186/1477-5956-9-52

**Published:** 2011-09-07

**Authors:** YuMei Zhang, JinMing Zhao, Yang Xiang, XiaoChun Bian, QiaoMei Zuo, Qi Shen, JunYi Gai, Han Xing

**Affiliations:** 1 National Center for Soybean Improvement, National Key Laboratory of Crop Genetics and Germplasm Enhancement, Nanjing Agricultural University, Nanjing 210095, P.R. China; 2 Guizhou Rapeseed Institute, Guizhou Academy of Agricultural Sciences, Guiyang 550008, P.R.China

## Abstract

**Background:**

*Phytophthora sojae *causes soybean root and stem rot, resulting in an annual loss of 1-2 billion US dollars in soybean production worldwide. A proteomic technique was used to determine the effects on soybean hypocotyls of infection with *P. sojae*.

**Results:**

In the present study, 46 differentially expressed proteins were identified in soybean hypocotyls infected with *P. sojae*, using two-dimensional electrophoresis and matrix-assisted laser desorption/ionization tandem time of flight (MALDI-TOF/TOF). The expression levels of 26 proteins were significantly affected at various time points in the tolerant soybean line, Yudou25, (12 up-regulated and 14 down-regulated). In contrast, in the sensitive soybean line, NG6255, only 20 proteins were significantly affected (11 up-regulated and 9 down-regulated). Among these proteins, 26% were related to energy regulation, 15% to protein destination and storage, 11% to defense against disease, 11% to metabolism, 9% to protein synthesis, 4% to secondary metabolism, and 24% were of unknown function.

**Conclusion:**

Our study provides important information on the use of proteomic methods for studying protein regulation during plant-oomycete interactions.

## Background

Soybean is one of the main sources of edible vegetable oil and high-protein livestock feed [1]. Phytophthora root and stem rot of soybeans, caused by the facultative pathogen *Phytophthora sojae*, is a serious disease. Each year it causes soybean damage estimated at one to two billion US dollars worldwide [2]. Breeding resistant cultivars is considered the most practical means of controlling this disease.

Two types of resistance to phytophthora root rot in soybeans have been described, partial and race-specific resistance. Partial resistance limits the spread of lesions in infected tissues. Race-specific resistance is monogenic and confers immunity or near immunity on the plant through a hypersensitive response. The different physiological pathotypes are governed by an *Rps *gene [3,4]. Currently, only 14 *Rps *genes at eight loci (*Rps1 *to *Rps8*) are known to confer soybean resistance to *P. sojae *and these have been designated and mapped to four molecular linkages: F, G, J, and N [5-8]. However, single *Rps *genes remain effective for 8-15 years [9]. The continuous introduction of stable *Rps *genes against *P. sojae *in soybean cultivars has resulted in the evolution of new pathogenic *P*. *sojae *races that can overcome the resistance conferred by these genes [10,11]. *P*. *sojae *is constantly evolving, and the number of physiological races of this oomycete pathogen is increasing rapidly [12]. Therefore, identifying and deploying new *Rps *genes is an essential part of soybean breeding programs.

With the development of second-generation sequencing technologies, microarray-based studies of soybean-*P. sojae *interactions are becoming more common. Many candidate genes presumably involved in regulating the expression of defense-related pathways for *Phytophthora *resistance in soybean have been identified [13,14]. High-throughput proteomic analysis is a powerful tool for studying changes in protein accumulation levels and posttranslational modifications [15]. In soybeans, the proteomic approach has been used to study many plant-pathogen interactions, including root hair infection with *Bradyrhizobium japonicum *[16], soybean mosaic virus [17], soybean cyst nematode [18], *P*. *sojae *[19], as well as symbiotic nitrogen fixation [20], and symbiotic microbe interactions [21].

Using the hypocotyl inoculation technique, Fan et al. [22] have mapped a novel allele at the *Rps1 *locus, and Sun et al. [23] discovered a novel gene in Yudou25. NG6255 is sensitive after inoculation with *P. sojae *[23]. In the present study, we set out to identify *P. sojae *resistance-related proteins in the Yudou25 and NG6255 cultivars of soybean. Yudou25 and NG6255 cultivars were inoculated with *P. sojae *and the differentially expressed proteins were analyzed and compared with those found in mock-inoculated controls. The results provide insights into the molecular basis of the differential responses of these two lines to this economically important plant pathogen.

## Results and discussion

### Contrasting phenotypes of Yudou25 and NG6255 in terms of resistance to *P. sojae *infection

**Figure 1:**
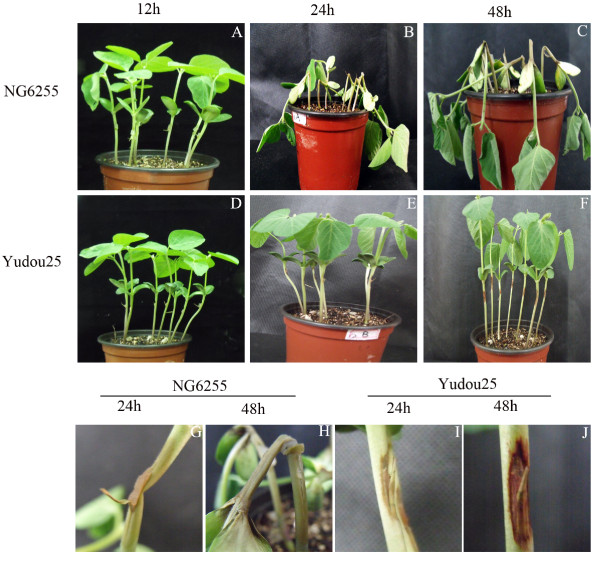
**Symptoms of soybeans 12, 24, and 48 h post-inoculation using the hypocotyl inoculation method**. A, B, and C show symptoms in NG6255. D, E, and F show symptoms in Yudou25. G, H, I, and J show close-up views of NG6255 (G, H) and Yudou25 (I, J) 24 and 48 h after inoculation.

Two soybean lines were challenged with the physiological race of *P. sojae *(PNJ1) isolated at the Jiangpu Farm, Nanjing Agricultural University during the growing season of 2006 [24]. We used the race-specific hypocotyl inoculation method to evaluate the two soybean lines for resistance against *P. sojae*, and examined the effects of the pathogen on them. The appearances of the two lines 12, 24 and 48 h after the PNJ1 challenge are shown in Figure 1. At 12 h after inoculation, the sensitive line, NG6255 (Figure 1A), and the resistant line, Yudou25 (Figure 1D) showed no physical difference. However, by 24 h and 48 h after inoculation, these lines showed remarkably different symptoms. Compared with the resistant line, Yudou25 (Figures 1E, and 1F), the sensitive line, NG6255 (Figures 1B, C) exhibited large, water-soaked lesions, as well as macerated and collapsed tissues. Magnified views of NG6255 and Yudou25, at 24 h and 48 h after inoculation, are shown in Figures 1G, H, I and 1J. Figures 1C and 1H, show serious wilting of the leaves and noticeable maceration of the stem of NG6255 48 h after inoculation with *P. sojae*, while Yudou25 showed only slight water-soaked lesions, indicating its higher tolerance to *P. sojae*.

### Proteomic patterns with and without *P. sojae *infection

**Figure 2:**
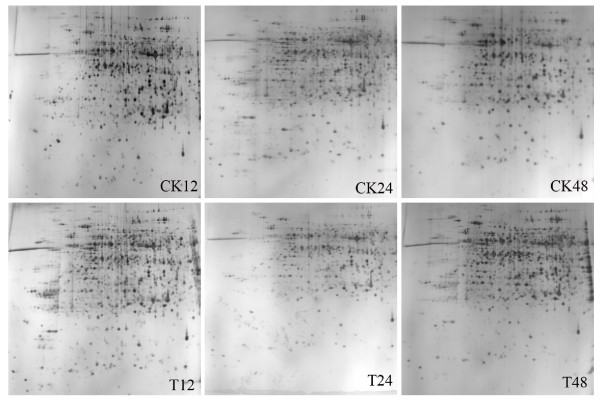
**Representative gel maps of Yudou25**. CK and T stand for mock- and PNJ1-inoculated soybean. Different inoculation times are shown on the bottom. The 2-DE was performed using 240 μg of protein, linear 17 cm IPG strips (pH 4-7), and 12% SDS-PAGE gels for second electrophoresis. Gels were stained with silver nitrate.

**Figure 3:**
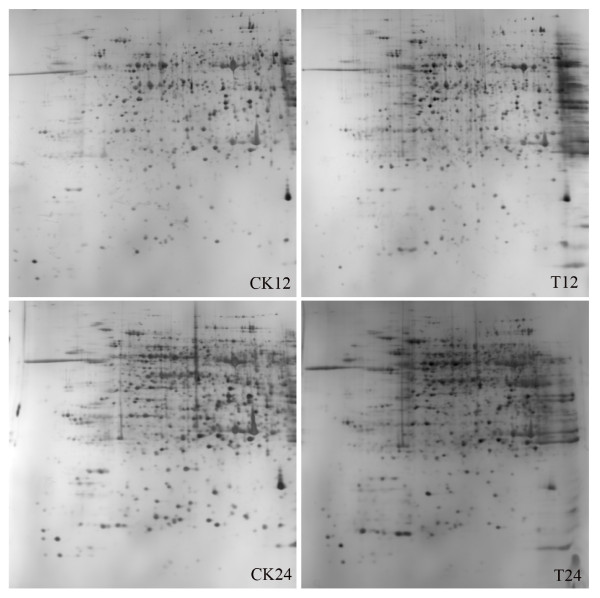
**Representative gel maps of NG6255**. CK and T stand for mock- and PNJ1-inoculated soybean. Different inoculation times are shown on the bottom. The 2-DE was performed using 240 μg of protein, linear 17 cm IPG strips (pH 4-7), and 12% SDS-PAGE gels for second electrophoresis. Gels were stained with silver nitrate.

Changes in the protein profiles of the hypocotyls infected with PNJ1 in the resistant line, Yudou25 (12, 24, and 48 h after inoculation), and the sensitive line, NG6255 (12 and 24 h after inoculation), were examined, using a 2-dimensional electrophoretic (2-DE) analysis of the total proteins present. We used immobilized pH gradient (IPG) strips (17 cm, pH 4-7) with relative molecular masses of 14-116 kDa and a silver stain. For each sample, the gel experiments were performed at least three times, and showed a high level of reproducibility (Figures 2 and 3 for Yudou25 and NG6255, respectively). The arrows and numbers in the figures refer to spots exhibiting significant changes in relative intensity after pathogen challenge. Typically, about 1,200 protein spots were detected on each silver-stained gel using PDQuest 7.1 software.

A total of 51 spots were observed in the two lines showing > 2-fold variation (*P *< 0.05), compared with the control at the same time point. However, five spots could not be identified. In the resistant line, 12 (spots 1, 4, 6, 7, 9, 10, 12, 16, 17, 21, 22, and 24) of the 26 spots were categorized as up-regulated, and 14 (spots 2, 3, 5, 8, 11, 13, 14, 15, 18, 19, 20, 23, 25, and 26) were down-regulated. Of these, a total of seven protein spots (spot 3, 5, 11, 13, 16, 19 and 20) showed a 2-fold change in at least two time points. Meanwhile, in the sensitive line, 11 (spots 29, 31, 32, 33, 34, 35, 36, 37, 44, 45, and 46) were up-regulated and nine (spots 27, 28, 30, 38, 39, 40, 41, 42 and 43) were down-regulated. Meanwhile, spots 28, 34, 37, 38, 43, 44 and 45 changed significantly 12 and 24 h after challenge with *P. sojae*.

In general, for most of the identified proteins, the experimental molecular weight (Mr) and isoelectric point (pI) were in reasonable agreement with the theoretical values of the matched proteins (see Additional file 1 and 2). However, differences between the experimental and theoretical values of Mr and pI for identities were also noticeable (see Additional file 1 and 2). For methionine synthase (spot 18), ribulose-1,5-bisphosphate carboxylase/oxygenase large subunit (spots 5, 15 and 26), the experimental Mr was markedly lower than the theoretical value (see Additional file 1). For phosphoglycerate kinase precursor-like protein (spot 8), ribulose-1,5-bisphosphate carboxylase small subunit (spot 25) and glyceraldehyde-3-phosphate dehydrogenase A subunit (spot 33), the theoretical pIs (7.68, 8.87 and 8.42, respectively) were beyond the pH range of the strips used. Wan and Liu [25] reported that the apparent Mr values predicted by sodium dodecyl sulfate-polyacrylamide gel electrophoresis (SDS-PAGE) had an error deviation of about ± 10% compared with the theoretical values. However, this phenomenon suggests that these proteins might be the partially degraded products of their intact proteins. Wan and Liu [25] also found that the observed molecular mass values of the 7 ribulose-1,5-bisphosphate carboxylase/oxygenase large subunits ranged from 19.14 to 30.18 kDa. This range is much smaller than the theoretical 52.79 kDa obtained by comparative proteomics using hydrogen peroxide stress tests in rice seedling leaves. Similar results were found in chilled rice seedling leaves, and further confirmed by Western blot analysis [26].

### Identification and functional classification of the differentially expressed proteins by MALDI-TOF/TOF

**Figure 4:**
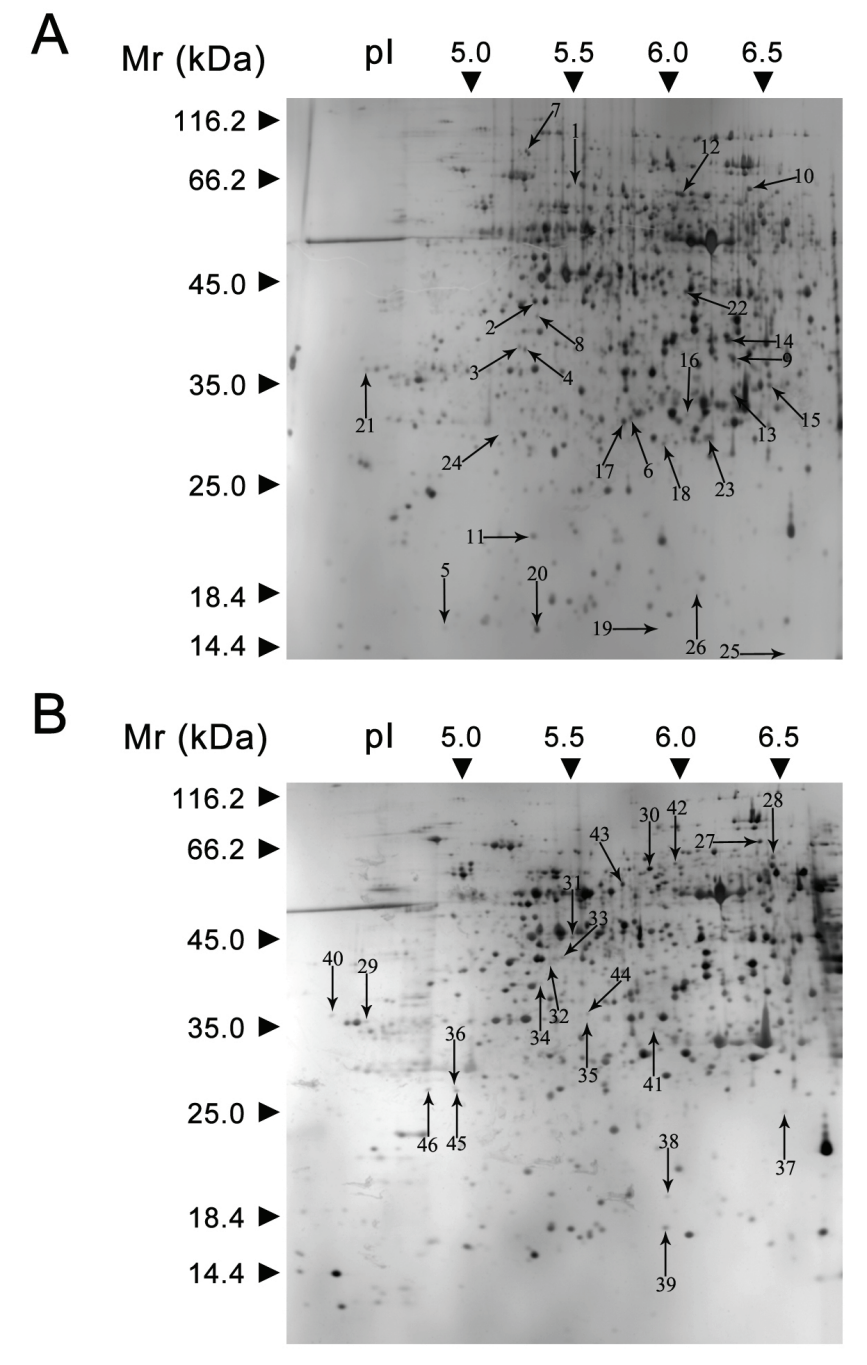
**Identification of 26 and 20 protein spots from Yudou25 (A) and NG6255 (B), respectively**. The numbers with arrows indicate the differentially expressed protein spots. pI and Mr are shown on the gels.

The identities of the proteins were determined using MALDI-TOF/TOF. Of the 51 proteins analyzed, 46 were identified, an identification success rate of approximately 83.8%. The 46 protein spots are shown in Figure 4A (Yudou25) and 4B (NG6255).

**Figure 5:**
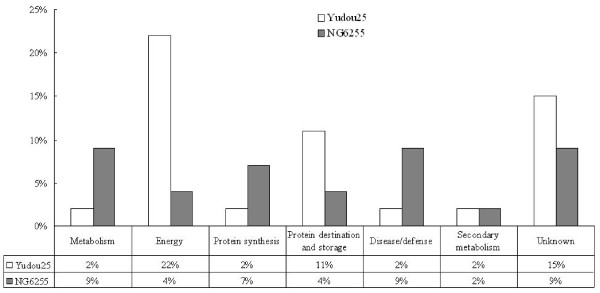
**Distribution of identified protein classes in Yudou25 and NG6255 using the methods of Bevan et al**. [27]. The percentages of the same functional class accounting for all the identified proteins from Yudou25 and NG6255 are represented in white and black, respectively.

Of these 46 spots, 35 were identified using the predicted sequences of the typical peptides from the NCBInr protein database (see Additional file 1 and 2). These 35 protein spots were placed in the current database as putative functional proteins, and the remaining 11 (spots 2, 3, 6, 16, 21, 22, 23, 29, 39, 40, and 44) were classified either as unknown or hypothetical proteins. Their identities were assigned by searching for their sequences using the BLASTP tool of the NCBInr protein database and their corresponding homologues with the highest similarity are listed in Additional file 3. All of these 11 proteins shared more than 70% positive identity with their homologues at the amino acid level, suggesting that they might have similar functions. All of the proteins identified were classified into 7 functional categories using the methods of Bevan et al. [27]: metabolism, energy, protein synthesis, protein destination and storage, defense against disease, secondary metabolism, or unknown. Figure 5 illustrates the distribution of these functional categories in Yudou25 and NG6255. In the resistant line, Yudou25, proteins related to energy dominated (22%). Those in the unknown, and protein destination/storage categories followed with 15% and 11%, respectively. In contrast, no category predominated in the sensitive line, NG6255. Proteins involved in metabolism, defense against disease and unknown each accounted for 9% of the total. The protein group related to synthesis followed, and the group related to secondary metabolism came last.

### Proteins involved in metabolism and energy regulation

Proteins involved in metabolism are essential for cell growth and maintenance. They accounted for 11% of the identified proteins in the present study. Pathogenic infections often affect certain common physiological processes, most importantly those to do with metabolism. In the present study, a number of the proteins affected at various times after pathogen challenge were those associated with energy pathways. These proteins accounted for 26% of all identified proteins, and were dominated by enzymes for glycolysis and photosynthesis.

In the resistant line, methionine synthase (spot 18) is involved in amino acid metabolism. The glycolysis-related proteins were phosphoglycerate kinase precursor-like protein (spot 8), aldose reductase (spot 9), glyceraldehyde-3-dehydrogenase C subunit (spot 14), and triosephosphate isomerase (spot 17). The proteins involved in photosynthesis were ribulose 1,5-bisphosphate carboxylase/oxygenase (spots 5, 11, 15, and 26), ribulose 1,5-bisphosphate carboxylase small subunit (spot 25), and NADP^+^-malic enzyme 1 (spot 12).

In the resistant line, the expression levels of the proteins involved in photosynthesis were down-regulated (see Additional file 1). Similar results were observed in pea plants (cv. Alaska) infected with plum pox virus (PPV, Sharka) [28]. Most of the changes in protein expression at the sub-cellular level produced by PPV infection were related to photosynthesis and carbohydrate metabolism.

Among the identified proteins, aldose reductase (spot 9) belongs to a superfamily of soluble NAD(P)(H) oxidoreductases. The superfamily reduces aldehydes and ketones, as well as producing ATP to protect seedlings. NADP^+^-Malic enzymes (spot 12) catalyze the oxidative decarboxylation of L-malate, producing pyruvate, CO_2_, and NAD(P)H in the presence of a divalent cation [29]. Spot 14 was additionally identified as GAPDH.

In the sensitive line, amino acid metabolism-related proteins included nitrite reductase (spot 28), glutamate-1-semialdehyde 2,1-aminomutase (spot 31), *S*-adenosylmethionine synthetase (spot 32), and isopropylmalate synthase (spot 42). The glycolysis-related proteins were hypothetical protein ARALYDRAFT_491252 (spot 30), and glyceraldehyde-3-dehydrogenase A subunit (spot 33).

*S*-Adenosylmethionine synthetase (spot 32) catalyzes the formation of *S*-adenosyl-Met from Met and ATP. Aside from its well-known role as a methyl donor in a myriad of transmethylation reactions, Ado-Met is involved in several essential functions for plant growth and development. These include being a precursor in ethylene biosynthesis, the propylamino group donor in the biosynthesis of the polyamines spermidine and spermine, and a key enzyme in lignin biosynthesis [30]. Spot 28 (nitrite reductase) was shown to be expressed at lower levels 12 h after inoculation in the sensitive line. Nitrate and nitrite reductase also utilize nitrite as a substrate. Nitric oxide (NO) is known to play a role in promoting programmed cell death (PCD) as a means of inhibiting pathogen attack. NO can act either as an antioxidant or promote PCD, depending on its concentration, and possibly on the timing as well as location of its production [31-33].

GAPDH was identified in both resistant and sensitive lines. In general, GAPDH is considered to be a house-keeping protein involved in basic catabolic cell processes and in numerous sub-cellular metabolic processes, including gene transcription, DNA replication, and endocytosis, among others. A previous study revealed that GAPDH activity is inhibited by H_2_O_2 _at the proteomic level. This finding suggests that GAPDH is a direct target of H_2_O_2 _and may have a role in mediating the signals of reactive oxygen species (ROS) in plants [34].

For example, when rice seedlings were exposed to high doses of H_2_O_2_, the small exogenous, stable, H_2_O_2 _molecule diffused into cells by cross-membrane-bound transport [35]. Some enzymes such as membrane-bound NADPH oxidase and apoplast-located amine oxidase were also activate to amplify endogenous H_2_O_2 _[36] and the intracellular H_2_O_2 _level in the rice leaves increased. Excessive H_2_O_2 _levels in the leaves caused an imbalance of the original redox homeostasis and elevated the oxidative intensity. This phenomenon leads to changes in the biomolecular metabolism.

Based on these results, we hypothesize that in soybean-*P. sojae *interaction, the host limits the spread of lesions in infected tissues mainly by mediating ROS signals and initiating PCD to ward off, or antagonize, the pathogen attack.

### Proteins involved in defense against disease

Overproduction of ROS is one of the first responses of plant cells to infection. ROS can also contribute to PCD, thereby serving as signal molecules for inducing local and systemic resistance [37]. Several proteins involved in detoxifying the free radicals generated during stress (e.g., pathogen challenge) were elevated after the *P. sojae*-resistant and *P. sojae*-sensitive lines were challenged with the pathogen.

In the resistant line, the proteins related to defense against disease were ascorbate peroxidase (spot 24). In the sensitive line, these proteins were ascorbate peroxidase (APx, spots 45, and 46), cytosolic ascorbate peroxidase 2 (spot 36) and lectin (spot 41).

Lectin (spot 41) was down-regulated in the sensitive line. Most plant lectins play a role in defending against different kinds of attacking organisms [38,39]. Galectins are released from outer membranes by enzymatic digestion of surface carbohydrates [40]. Soluble plant lectins are also possibly linked to a region near the plasma membrane through interactions with surface glycoproteins. The secretion of plant lectins into the medium may play a role in defense or symbiosis via bacterial or fungal binding and subsequent agglutination. Similar results are shown in proteome analysis to flood stress [41] and NaCl stress [42] in soybean roots and hypocotyls.

APXs, components of the ascorbate-glutathione cycle are widely distributed in plant cytosol, mitochondria, chloroplasts and other cellular compartments [33,43]. In these organelles, ROS scavenging is required because of the importance of the ascorbate-glutathione cycle in maintaining cellular ROS homeostasis. The elevated levels of ascorbate peroxidase observed in this study possibly led to an amelioration of infection-induced oxidative stress. The intensities of the spots identified as APx increase in both resistant and sensitive lines, suggesting that differences in antioxidant responses may have important roles in determining the outcome of an infection. The role of APx in defence against *P. sojae *needs further study.

### Proteins involved in protein synthesis, destination, and storage

A total of 11 identified proteins (24%) that responsed to *P. sojae *infection (see Additional file 1 and 2) were divided into two functional groups. The first group consisted of four proteins active in protein synthesis, and the second comprised seven proteins related to destination and storage.

In the resistant line, the proteins active in synthesis, and protein destination and storage (spot 7, translation elongation factor EF-G) accounted for 2% and 13% of the total, respectively.

In the sensitive line, those involved in protein synthesis (7%) were 60S acidic ribosomal protein P0 (spot 34), hypothetical protein SORBIDRAFT_0525s002010 (spot 35), and eukaryotic translation initiation 5A-2 (spot 38). Those related to protein destination and storage (4%) were HSP STI (spot 27) and 31 kDa glycoprotein (spots 37).

HSPs are responsible for protein refolding and assembly, thereby conferring protection. HSPs are active in diverse cellular processes as molecular chaperones [44]. The intensity of HSP70 (spot 1) and LOC100285569 (spot 10) were up-regulated after challenge in the resistant line. In contrast, HSP STI (spot 27) was down-regulated in the sensitive line. Some proteins involved in synthesis were up-regulated, but others with similar functions, were down-regulated. These results suggested that these proteins had different regulatory pathways.

The 31 kDa glycoproteins (spots 13, 19, 20) in the resistant line were down-regulated, but up-regulated in the sensitive line (spot 37). The stem 31 kDa glycoprotein precursor may function as a somatic storage protein during early seedling development, and accumulates mainly in the stems of developing soybean seedlings [45,46]. Similar results occur in proteome analysis of soybean leaves, hypocotyls and roots under salt stress [42,47] and flood stress [41].

### Proteins involved in secondary metabolism

Secondary metabolites are distinct from the components of intermediary (primary) metabolism in that they are not generally essential for the basic metabolic processes of the plant. However, they do play a crucial role in many plant processes, and their syntheses should be intensified during pathogen infection. Important compounds such as lignin, suberin, cell wall-bound phenolics, and flavonoids derive their building units from the phenylpropanoid pathway. These compounds are essential in defense and tissue reconstruction [48-50].

Spot 4 was identified as thiamin biosynthetic enzyme in the resistant line Yudou25. This protein was up-regulated at 48 h after pathogen challenge.

A recent study suggested that thiamin can directly act as an antioxidant [51]. The association between thiamin-dependent enzymes and oxidative stress may also indicate the activity of several cofactors for thiamin under stress conditions. Ahn et al. [52,53] reported that thiamin treatment induced systemic acquired resistance in plants. Meanwhile, the exogenous application of thiamin has been shown to counteract the harmful effects of salinity on growth [54], as well as to confer resistance to fungal, bacterial, and viral infections in rice (*Oryza sativa*), *Arabidopsis*, and certain vegetable crop species [55]. The role of thiamin biosynthetic enzymes in mediating the response to *P. sojae *warrants further investigation.

Spot 43 was also involved in secondary metabolism in the sensitive line. Myo-inositol-1-phosphate synthase (MIPS) catalyzes the conversion of D-glucose 6-phosphate to 1-L-myo-inositol-1-phosphate. This conversion is the first, and rate-limiting step in the biosynthesis of all inositol-containing compounds, including phospholipids, either directly or by salvage [56].

Iqbal et al. [57] reported that MIPS plays an important role in initiating and/or maintaining the defense response of soybean to *F. solani *infection, via Ca^2+ ^signaling. MIPS1 has a significant impact on myo-inositol levels that is critical for maintaining levels of ascorbic acid, phosphatidylinositol, and ceramides. These compounds regulate growth, development, and cell death in *A. thaliana *[58]. Other studies have shown that introgression of *PcINO1 *gene from *Porteresia coarctata *(Roxb.) Tateoka, confers salt tolerance on transgenic tobacco plants. This gene codes a novel salt-tolerant, L-myo-inositol 1-phosphate synthase (MIPS) protein [59,60]. In our study, the intensity of MIPS was similarly down-regulated, which suggested its vital role in *P. sojae *infection.

### Proteins of unknown identity

Eleven of the proteins isolated (24% of the total) were classified as unknown, including seven (15%) in the resistant line, Yudou25, and four (9%) in the sensitive line, NG6255. Their homologues based on the NCBI database by BLASTP are shown in Additional file 3, and the homologues with the highest homology are also shown in Additional file 3.

The homologues of the unknown *P. sojae *responsive proteins were found to be involved in diverse biological processes, from signal transduction (spots 2 and 16), defense against disease (spots 3 and 39), metabolism and energy regulation (spots 6, 23 and 44), protein synthesis (spots 21 and 29), protein destination and storage (spot 40) and secondary metabolism (spot 22).

Spot 2 belongs to an adenosine kinase-like protein. Spot 16 had the highest homology to indole-3-glycerol phosphate lyase (IGL2). IGL cleaves indole-3-glycerol phosphate (IGP) to form indole and glyceraldehydes-3-phosphate. Indole, which is part of the cocktail, is produced by an enzyme recruited from primary metabolism, and can function either as a volatile signal or be converted by specific cytochrome P450 enzymes into benzoxazinoids, which function as important defense chemicals [61]. IGL2 is involved in auxin-mediated signal transduction. The interaction of auxin with its cellular receptors for IGL2 triggers a cascade of events resulting in response including the modification of cell wall components such as lipids, and in altering the orientation of cell wall polysaccharides [62]. In a previous study, the application of indole-3-acetic acid decreased the incidence of disease caused by the tomato pathogen *Fusarium oxysporum lycopersici *and increased plant-growth [63]. The proteome-level results suggested ROS-mediated auxin signaling had a role in the *Brassica napus*-*Alternaria brassicae *interaction [64].

Spot 3 and 39 were highly homologous to globulin and peroxiredoxin, respectively. These two protein spots may take part in denfense against disease. Spot 40 was identified as Nascent polypeptide-associated complex subunit alpha-like protein 2 (NACA2), which take part in correct orientation of ribosomal nascent polypeptides with directional factors. The decreasing expression of spot 40 identified from the sensitive line, NG6255, could cause inaccurate direction, or indirection, for most nascent proteins, resulting in the proteins becoming dysfunctional, or not being delivered to the right place to function properly. Spot 44 (gi|255638532) belongs to the Aldose 1-epimerase family of proteins, which are key enzymes in carbohydrate metabolism. These enzymes catalyze the inter-conversion of the α- and β- anomers of hexose sugars such as glucose and galactose.

Lignification processes are important in protecting plants from pathogen attack. The closest homologue to S-adenosyl-L-methionine:caffeic acid 3-o-methyltransferase (COMT) was spot 22. This transferase catalyzes the conversion of caffeic acid to ferulic acid, a key step in the biosynthesis of lignin monomers [65]. These products are the intermediates for lignin formation, and the down-regulation of this enzyme may indicate decreased cell wall lignification. Guo et al. [66] reported that strong down-regulation of COMT resulted in decreasing lignin content in transgenic alfalfa plants harboring COMT. In the present study, spot 22 was up-regulated in the resistant line, Yudou25, indicating that enhanced lignin biosynthesis may play a role in mediating the effects observed during soybean-*P. sojae *interaction, and should be further investigated.

## Conclusion

Proteomic analysis has the potential to provide significant insights into the molecular events that occur during plant-pathogen interactions. In the present study, we investigated the proteome-level changes that occur during soybean-*P. sojae *interaction using 2-DE and tandem MS. The proteins showing two-fold changes in intensities are related to biochemical processes that may be differentially altered at various time points after pathogen challenge. Whereas some enzymes/proteins are involved in glycolysis and photosynthesis, others are involved in protein synthesis, destination and storage, defense against disease, as well as in secondary metabolism. The results from the present study offer insights into the repertoire of mechanisms used by *P. sojae *during its infection and colonization of a host.

## Methods

### *P. sojae *isolate and plant material

PNJ1 was originally isolated from the diseased tissues of soybean and soil samples in Nanjing in 2006 [24]. The isolate was established from single oospores, and was tested for virulence pathotype using the hypocotyl inoculation method on 14 differential cultivars. The virulence formula of PNJ1 is 1d, 2, 3b, 3c, 4, 6, 7. The isolate was maintained on V8-juice agar plates, in the dark, at 25 °C.

The plant materials used included cultivars Yudou25 and NG6255, previously screened from Huanghuai valley cultivars via the hypocotyl inoculation technique [67]. Yudou25 is a PNJ1-resistant cultivar, whereas NG6255 is sensitive to PNJ1 [23]. Their seedlings were grown on a commercially available 3:1 mixture of vermiculite and peat moss, under controlled conditions (16 h light, 8 h dark; 28 °C at day, 22 °C at night; 60% relative humidity; light intensity 600 μmol photons m^-2 ^s^-1^).

The soybean lines were evaluated using the hypocotyl inoculation method [68] with minor modifications. Mycelia from 7-day-old V8 cultures were inoculated into an incision in the hypocotyls of the seedlings when the cotyledons were fully opened. The seedlings were then placed in a mist chamber (90% relative humidity) at 25 °C with a 16:8 h light:dark cycle. The control plants were wounded but not inoculated with the mycelia. The hypocotyl section samples were collected from 10 mm below and above the incision after inoculation.

### Protein sample preparation

Soybean stem proteins were extracted using a modified trichloroacetic acid/acetone procedure following Natarajan et al. [69], with minor modifications. The precipitated proteins were washed with ice-cold acetone containing 10 mM dithiothreitol (DTT) and 1 mM phenylmethanesulfonyl fluoride to remove pigments and lipids until the supernatant was colorless. The pellet was vacuum dried, resuspended in resolubilization solution {7 M urea, 2 M thiourea, 4% [(3-cholamidopropyl) dimethylammonio] propanesulfonic acid (CHAPS), 50 mM DTT, 0.5% Bio-Lyte 4/7 ampholyte}, and sonicated to extract the proteins. After incubation at 25 °C for 4 h, the suspension was centrifuged at 15,000 × *g *for 30 min at 4 °C to remove the insoluble material. The protein concentration of the final supernatant was measured following Bradford [70], using bovine serum albumin as the standard.

### 2-DE, gel staining, and image analysis

One-DE was performed in a Bio-Rad PROTEAN IEF Cell. The protein extract was diluted to a final concentration of 800 μg/ml with an IEF rehydration solution [7 M urea, 2 M thiourea, 4% CHAPS (*w/v*), 50 mM DTT, and 0.5% (*v/v*) IPG buffer (pH 4-7)]. After centrifugation for 15 min at 10,000 × *g*, 300 μl of supernatant was loaded onto a commercially available precast IPG strip with a linear 17 cm pH 4-7 gradient and then actively rehydrated at 50 V for 13 h at 20 °C. Focusing was performed on a Bio-Rad PROTEAN IEF Cell under the following conditions: 100 V for 1 h, 500 V for 1 h, 1000 V for 2 h, 8000 V for 4 h, and then 8000 V for 7.5 h. Approximately 60,000 Vh was achieved. Before SDS-PAGE, the strips were equilibrated for 15 min in 10 ml of a reducing equilibration buffer [6 M urea, 0.375 M Tris-HCl (pH 8.8), 2% (*w/v*) SDS, 20% glycerol (*v/v*), and 2% (*w/v*) DTT]. The strips were subsequently placed for another 15 min in alkylating equilibration buffer containing 2.5% (*w/v*) iodoacetamide instead of 2% DTT.

The second dimension was run on a 12% polyacrylamide SDS gel using an EttanTM DALT SIX System (GE Healthcare). The electrophoresis was carried at 20 °C and 1.0 W/gel for 40 min and then at 10 W/gel until the dye front reached about 1 cm from the bottom of the gel.

The gels were stained with silver nitrate as described by Yan et al. [71]. The silver-stained 2-DE gels were digitized with a Versdoc 3000 scanner (Bio-Rad), and analyzed using PDQuest software (version 7.1, Bio-Rad, Hercules, CA, USA). The images were properly cropped and optimized before performing the gel-to-gel matching of the standard protein maps. Before spot matching, one of the gel images was selected as a reference gel. The spot detection parameters were optimized by checking different protein spots in certain regions of the gel, which were then automatically detected. Visual inspection followed to remove or add undetected spots. Spot detection was refined by a manual spot edition where needed. The amount of a protein spot was expressed as the volume of that spot, defined as the sum of the intensities of all the pixels that make up the spot. To correct the variability due to the loading and gel staining, as well as to reflect the quantitative changes in the intensity of the protein spots, the spot volumes were normalized as a percentage of the total volume of all the spots in the gel. The resulting data from the image analyses were transferred to PDQuest software for querying protein spots that showed quantitative and qualitative variations. Triplicate gels were used for each sample. Only those changes that were reproducible and showed at least two-fold increase/decrease, as well as being statistically significant (using Student's *t*-test, at *P *< 0.05) were considered to be differentially expressed protein spots.

### In-gel digestion, mass spectrometry analysis, and database search

Differentially expressed proteins were manually selected and excised for protein identification. The in-gel digestion of protein spots was carried out following Gharahdaghi et al. [72]. The samples were analyzed with a 4800 MALDI-TOF/TOF Proteomics Analyzer (Applied Biosystems, USA). A combined search (MS plus MS/MS) were performed using GPS Explorert™ software (version 3.5, Applied Biosystems, USA). The TOF spectra were recorded in positive ion reflector mode with a mass range from 800 to 4000 Da. About eight subspectra with 60 shots per subspectrum were accumulated to generate one main TOF spectrum. Data were searched on the Internet using a Mascot search engine (Matrix Science, Ltd., London, UK) against all entries in the NCBInr database (version 841369 sequences). Viridiplantae (Green Plants) was selected as the taxonomic category. All peptide masses were assumed monoisotopic and [M+H]^+^. The other parameters used for the search were: enzyme of trypsin; one missed cleavage site at most; fixed modifications of cysteines carbamidomethylation and variable modifications of methionine oxidation; peptide tolerance within 50 ppm, and MS/MS tolerance of 0.2 Da. The confidence in the peptide mass fingerprinting matches (*P *< 0.05) was based on the MOWSE score and confirmed by the accurate overlapping of the matched peptides with the major peaks of the mass spectrum. Only significant hits, as defined by a MASCOT probability analysis (*P *< 0.05), were accepted.

## Competing interests

The authors declare that they have no competing interests.

## Authors' contributions

BXC and ZQM carried out sample collection and protein extraction, ZYM and ZJM carried out 2-DE, image acquisition and data analysis. XY and SQ helped in manuscript revision. GJY and XH conceived, designed and implemented this study. All authors read and approved the final manuscript.

## Supplementary Material

Additional file 1 **Identification of 26 proteins from the resistant line Yudou25 at various times after challenge with the pathogen**. a) Spot No, Spot number; b)Names and species of proteins obtained via the MASCOT software from the NCBInr database; c) Accession No, Accession number; d)The sequences of all the identified peptides with the corresponding ion score in brackets that were matched based on the MS/MS patterns; e)MOWSE score probability (protein score) for the entire protein and for ions complemented by the percentage of the confidence index (C.I.); f)SC, Sequence coverage; g)MP/UMP indicate the number of matched and unmatched peaks for the PMF data, respectively; h) Theor. Mr/pI shows theoretical molecular weight and pH isoelectric; i)Exp. Mr/pI shows experimental molecular weight and isoelectric point; j)Fold change was calculated from pathogen-challenged tissue over the control gels, which '-' stands for down-regulated. Click here for file

Additional file 2 **Identification of 20 proteins from the sensitive line NG6255 at various times after challenge with the pathogen**. a) Spot No, Spot number; b)Names and species of proteins obtained via the MASCOT software from the NCBInr database; c) Accession No, Accession number; d)The sequences of all the identified peptides with the corresponding ion score in brackets that were matched based on the MS/MS patterns; e)MOWSE score probability (protein score) for the entire protein and for ions complemented by the percentage of the confidence index (C.I.); f)SC, Sequence coverage; g)MP/UMP indicate the number of matched and unmatched peaks for the PMF data, respectively; h) Theor. Mr/pI shows theoretical molecular weight and pH isoelectric; i)Exp. Mr/pI shows experimental molecular weight and isoelectric point; j)Fold change was calculated from pathogen-challenged tissue over the control gels, which '-' stands for down-regulated. Click here for file

Additional file 3 **The corresponding homologues of the eleven unknown proteins**. BLASTP(NCBI) was used to search the homologues of the unknowm proteins in additional file 1 and 2. The homologues with the highest homology are shown. a) The accession number of the unknown proteins in additional file 1 and 2; b) The accession number of the homologues; c) The extent to which two amino acid sequences are invariant; d) The similarities based on the scoring matrix used. Click here for file
