# Supplementary material for: Proteomics study of changes in soybean lines resistant and sensitive to Phytophthora sojae
Source: Proteome Sci. 2011 Sep 7;9:52. doi: 10.1186/1477-5956-9-52 (PMC3180303; doi:10.1186/1477-5956-9-52)
Supplement: Additional file 1 — Identification of 26 proteins from the resistant line Yudou25 at various times after challenge with the pathogen. a) Spot No, Spot number; b)Names and species of proteins obtained via the MASCOT software from the NCBInr database; c) Accession No, Accession number; d)The sequences of all the identified peptides with the corresponding ion score in brackets that were matched based on the MS/MS patterns; e)MOWSE score probability (protein score) for the entire protein and for ions complemented by the percentage of the confidence index (C.I.); f)SC, Sequence coverage; g)MP/UMP indicate the number of matched and unmatched peaks for the PMF data, respectively; h) Theor. Mr/pI shows theoretical molecular weight and pH isoelectric; i)Exp. Mr/pI shows experimental molecular weight and isoelectric point; j)Fold change was calculated from pathogen-challenged tissue over the control gels, which '-' stands for down-regulated. [file 1477-5956-9-52-S1.DOC]

Identification of 26 proteins from the resistant line Yudou25 at various times after challenge with the pathogen

| Spot  No.^a)^ | Protein  Name^b)^ | Accession  No.^c)^ | Matched peptide  Sequences (Ion Score)^d)^ | Score/  Threshhold  ^e)^ | SC  (%)^f)^ | MP/  UMP^g)^ | Theor.  Mr/pI^h)^ | Exp.  Mr/pI^i)^ | Fold Change^j)^ | | |
| --- | --- | --- | --- | --- | --- | --- | --- | --- | --- | --- | --- |
|  |  |  |  |  |  |  |  |  | 12h | 24h | 48h |
| **Functional category 01 metabolism** | | | | | | | | | | | |
| 18 | methionine synthase [Glycine max] | gi|33325957 | LIRNELAK(53)  NILWVNPDCGLK(63)  AGITVIQIDEAALR(52)  IPPTEEIADRINK(63)  YGAGIGPGVYDIHSPR(105) | 334/42 | 8 | 25/57 | 84.4/5.93 | 28.0/6.00 | -1.06 | -3.32 | 1.01 |
| **Functional category 02 energy** | | | | | | | | | | | |
| 5 | ribulose-1,5-bisphosphate carboxylase/oxygenase large subunit  [Glycine max] | gi|91214125 | VALEACVQAR(51)  LSGGDHVHAGTVVGK(88) | 241/42 | 10 | 16/49 | 53.0/6.00 | 17.0/4.95 | -3.19 | -4.31 | -1.45 |
| 8 | Phosphoglyceratekinase precursor  [Solanum tuberosum] | gi|3328122 | No hit | 46/42 | 5 | 16/114 | 50.6/7.68 | 41.5/5.29 | -1.30 | -5.43 | -4.86 |
| 9 | aldose reductase  [Digitalis purpurea] | gi|13160399 | FFELNTGAK(54) | 94/43 | 5 | 8/76 | 35.1/5.64 | 35.8/6.38 | 1.18 | 1.14 | 2.26 |
| 11 | ribulose-1,5-bisphosphate carboxylase/oxygenase large subunit [Glycine max] | gi|290586534 | DTDILAAFR(78)  LTYYTPDYETK(74)  LEDLRIPTAYIK(51)  TFQGPPHGIQVER(101)  LTYYTPDYETKDTDILAAFR(165) | 504/44 | 20 | 14/101 | 26.3/6.93 | 21.8/5.30 | -1.08 | -4.28 | -4.77 |
| 12 | NADP^+^-malic enzyme 1  [Arabidopsis thaliana] | gi|15225262 | LLNDEFYIGLR(73) | 104/44 | 3 | 14/88 | 64.5/6.32 | 64.4/6.12 | 2.10 | 1.28 | 1.68 |
| 14 | glyceraldehyde-3-dehydrogenase  C subunit [Glycine max] | gi|74475508 | YDSVHGHWK(43)  HHDVTVKDEK(83)  AASFNIIPSSTGAAK(72)  LVSWYDNEWGYSSR(85)  GILGYTEDDVVSTDFIGDSR(43) | 325/40 | 20 | 26/66 | 36.8/6.72 | 33.8/6.39 | -1.29 | -4.27 | 1.31 |
| 15 | ribulose-1,5-bisphosphate carboxylase/oxygenase large subunit [Glycine max] | gi|91214125 | VALEACVQAR(47)  DNGLLLHIHR(79)  LSGGDHVHAGTVVGK(100)  GGLDFTKDDENVNSQPFMR(102) | 427/44 | 16 | 33/41 | 53.0/6.00 | 33.8/6.58 | -1.11 | -3.49 | 1.12 |
| 17 | triosephosphate isomerase [Glycine max] | gi|48773765 | VIACIGETLEQR(59)  WVHDNVSAEVAASVR(129)  VATPAQAQEVHADLRK(74) | 276/41 | 23 | 17/85 | 27.4/5.87 | 29.5/5.79 | 2.09 | -1.14 | 1.13 |
| 25 | ribulose-1,5-bisphosphate  carboxylase small subunit rbcS1  [Glycine max] | gi|10946375 | IIGFDNVR(61)  LPMFGCTDASQVLK(113)  QVQCISFIAYKPPGF(133)  SPGYYDGRYWTMWK(77) | 458/43 | 32 | 20/55 | 20.2/8.87 | 12.6/6.65 | 1.33 | -2.04 | 1.12 |
| 26 | ribulose-1,5-bisphosphate carboxylase/oxygenase large  subunit [Muscoflorschuetzia pilmaiquen] | gi|20531004 | LSGGDHVHAGTVVGK(62) | 134/44 | 8 | 17/14 | 47.0/6.30 | 18.4/6.16 | 1.48 | -7.11 | -1.14 |
| **Functional category 05 protein synthesis** | | | | | | | | | | | |
| 7 | translation elongation factor  EF-G[Glycine max] | gi|402753 | No hit | 66/42 | 9 | 23/18 | 77.9/5.04 | 89.8/5.23 | -1.71 | 2.02 | -1.80 |
| **Functional category 06 protein destination and storage** | | | | | | | | | | | |
| 1 | Heat shock 70 kDa protein,  Mitochondrial  [Phaseolus vulgaris] | gi|399940 | No hit | 88/72 | 35 | 27/103 | 72.7/5.95 | 60.8/5.40 | 4.25 | 1.18 | 1.14 |
| 10 | LOC100285569 [Zea mays] | gi|226531388 | ALELDDEDISYLTNR(104) | 144/43 | 4 | 9/86 | 65.8/6.26 | 65.0/6.44 | -1.63 | 1.56 | 2.49 |
| 13 | 31kD glycoprotein [Glycine max] | gi|226866 | LAVEAHNIR(67)  TVNQQAFFYASER(109)  TIPEECVEPTKDYINGEQFR(89) | 285/42 | 19 | 17/70 | 29.8/6.72 | 32.8/6.39 | 1.51 | -3.31 | -2.76 |
| 19 | Stem 31 kDa glycoprotein [Glycine max] | gi|1351033 | EYIHGEQYR(55)  TVNQQAYFYAR(90) | 145/42 | 6 | 9/56 | 33.2/8.59 | 14.6/5.97 | 1.22 | -16.23 | -2.94 |
| 20 | 31kD glycoprotein [Glycine max] | gi|226866 | LAVEAHNIR(62)  TVNQQAFFYASER(101)  TIPEECVEPTKDYINGEQFR(65) | 228/40 | 16 | 13/77 | 29.8/6.72 | 16.4/5.22 | 1.24 | -3.63 | -3.68 |
| **Functional category 11 disease/defense** | | | | | | | | | | | |
| 24 | ascorbate peroxidase  [Glycine max] | gi|310561 | LAWHSAGTFDKGTK(72) | 114/40 | 17 | 11/18 | 27.1/5.51 | 29.3/5.10 | 2.11 | -1.12 | -1.90 |
| **Functional category 20 secondary metabolism** | | | | | | | | | | | |
| 4 | thiamin biosynthetic enzyme  [Glycine max] | gi|6552391 | no hit | 282/72 | 15 | 12/34 | 36.7/5.77 | 37.1/5.23 | 1.02 | 1.07 | 3.08 |
| **Functional category 12 unknown** | | | | | | | | | | | |
| 2 | unknown [Glycine max] | gi|255634788 | LYPVILLPK(54)  QKPIEECVR(58)  SLVANLSAANCYK(118)  AQGWETDNVEEIALK(140)  SEHLTRPENWALVEK(54)  VLPYMDYVFGNETEAR(104) | 528/41 | 22 | 26/104 | 38.2/5.32 | 42.5/5.31 | -3.09 | 1.06 | 1.11 |
| 3 | unknown [Glycine max] | gi|255641502 | IYGLDKDGVEK(74)  NSTTREEVVVK(45)  VGYVLQGNDGVAGMALR(118)  LVLQPQGFAIPHYSDISK(131) | 368/43 | 25 | 14/92 | 24.7/5.01 | 37.5/5.20 | -5.24 | -2.32 | 1.29 |
| 6 | unknown [Glycine max] | gi|255628027 | No hit | 59/43 | 8 | 13/42 | 24.4/5.58 | 40.5/5.82 | -1.56 | 1.14 | 2.92 |
| 16 | unknown [Glycine max] | gi|255634482 | No hit | 92/72 | 42 | 11/69 | 28.3/5.53 | 31.0/6.14 | 2.30 | 1.01 | 2.01 |
| 21 | unknown [Glycine max] | gi|255627339 | KLDEYLLPR(76)  SVQMEGLLWGASK(113)  SSVLLDVKPWDDETDMK(53) | 291/43 | 21 | 16/32 | 25.1/4.46 | 35.3/4.55 | 1.10 | 2.09 | 1.13 |
| 22 | unknown [Glycine max] | gi|255644467 | ILETYTGFEGLK(93)  SALELDLLEIIAK(89)  AGPGVHLSPSDIASR(69) | 315/41 | 21 | 29/65 | 40.3/5.74 | 45.0/6.14 | -1.44 | 1.14 | 2.01 |
| 23 | unknown [Glycine max] | gi|255641005 | TVNQQAYFYAK(100)  LILRDPSEYSGK(58)  NLKEVGFNTWEK(70)  IIGNVGDQWSDLLGSNK(83) | 346/42 | 21 | 16/49 | 29.0/6.34 | 28.8/6.27 | -2.90 | -1.07 | 1.01 |

a) Spot No, Spot number;

b)Names and species of proteins obtained via the MASCOT software from the NCBInr database;

c) Accession No, Accession number;

d)The sequences of all the identified peptides with the corresponding ion score in brackets that were matched based on the MS/MS patterns;

e)MOWSE score probability (protein score) for the entire protein and for ions complemented by the percentage of the confidence index (C.I.);

f)SC, Sequence coverage;

g)MP/UMP indicate the number of matched and unmatched peaks for the PMF data, respectively;

h) Theor. Mr/pI shows theoretical molecular weight and pH isoelectric;

i)Exp. Mr/pI shows experimental molecular weight and isoelectric point;

j)Fold change was calculated from pathogen-challenged tissue over the control gels, which ‘-’ stands for down-regulated.
