# Supplementary material for: Proteomics study of changes in soybean lines resistant and sensitive to Phytophthora sojae
Source: Proteome Sci. 2011 Sep 7;9:52. doi: 10.1186/1477-5956-9-52 (PMC3180303; doi:10.1186/1477-5956-9-52)
Supplement: Additional file 2 — Identification of 20 proteins from the sensitive line NG6255 at various times after challenge with the pathogen. a) Spot No, Spot number; b)Names and species of proteins obtained via the MASCOT software from the NCBInr database; c) Accession No, Accession number; d)The sequences of all the identified peptides with the corresponding ion score in brackets that were matched based on the MS/MS patterns; e)MOWSE score probability (protein score) for the entire protein and for ions complemented by the percentage of the confidence index (C.I.); f)SC, Sequence coverage; g)MP/UMP indicate the number of matched and unmatched peaks for the PMF data, respectively; h) Theor. Mr/pI shows theoretical molecular weight and pH isoelectric; i)Exp. Mr/pI shows experimental molecular weight and isoelectric point; j)Fold change was calculated from pathogen-challenged tissue over the control gels, which '-' stands for down-regulated. [file 1477-5956-9-52-S2.DOC]

Identification of 20 proteins from the sensitive line NG6255 at various times after challenge with the pathogen

| Spot  No.^a)^ | Protein  Name^b)^ | Accession  No.^c)^ | Matched peptide  Sequences (Ion Score)^d)^ | Score/  Threshhold  ^e)^ | SC  (%)^f)^ | MP/  UMP ^g)^ | Theor.  Mr/Pi^h)^ | Exp.  Mr/pI^i)^ | Fold change^j)^ | |
| --- | --- | --- | --- | --- | --- | --- | --- | --- | --- | --- |
|  |  |  |  |  |  |  |  |  | 12h | 24h |
| **Functional category 01 metabolism** | | | | | | | | | | |
| 28 | nitrite reductase [Glycine max] | gi|1906002 | AVLEAYRDLGFR(43)  DGFWVLKEEYR(44) | 149/43 | 7 | 31/72 | 67.6/6.95 | 62.6/6.48 | -3.46 | -2.10 |
| 31 | Glutamate-1-semialdehyde 2,1-aminomutase, chloroplastic [Glycine max] | gi|1170031 | No hit | 89/42 | 10 | 19/95 | 49.8/5.67 | 46.2/5.53 | 3.03 | -1.12 |
| 32 | S-adenosylmethionine synthetase [Glycine sojae] | gi|201085690 | No hit | 72/72 | 27 | 13/46 | 43.4/5.74 | 42.1/5.46 | 3.56 | 1.49 |
| 42 | isopropylmalate synthase, putative [Ricinus communis] | gi|255579410 | No hit | 118/72 | 26 | 17/113 | 69.0/6.41 | 62.0/6.02 | -2.95 | 1.13 |
| **Functional category 02 energy** | | | | | | | | | | |
| 30 | hypothetical protein  [Populus trichocarpa] | gi|224060560 | ILNDETFAK(58)  VKDGIPHLTK(43)  GGVIDEDALVR(82)  IGDIPAVEEFVFLKL(56) | 306/42 | 9 | 15/90 | 63.7/6.26 | 60.7/5.88 | -3.41 | 1.46 |
| 33 | glyceraldehyde-3-phosphate dehydrogenase A subunit  [Glycine max] | gi|77540210 | VVDLADIVANKWK(50) | 98/41 | 12 | 29/78 | 43.5/8.42 | 43.3/5.48 | 5.20 | 1.30 |
| **Functional category 05 protein synthesis** | | | | | | | | | | |
| 34 | 60S acidic ribosomal protein P0  [Glycine max] | gi|1710587 | No hit | 50/40 | 5 | 15/67 | 34.2/5.15 | 38.3/5.40 | 11.14 | 2.24 |
| 35 | hypothetical protein SORBIDRAFT_0525s002010  [Sorghum bicolor] | gi|253760744 | No hit | 79/72 | 35 | 17/67 | 38.3/5.57 | 34.5/5.61 | 16.18 | 1.34 |
| 38 | eukaryotic translation initiation factor 5A-2 [Glycine max] | gi|217038832 | TYPQQAGTIR(55)  CHFVGIDIFTAK(92)  KLEDIVPSSHNCDVPHVNR(43) | 217/41 | 26 | 12/60 | 17.7/5.60 | 19.6/5.99 | -2.45 | -2.21 |
| **Functional category 06 protein destination and storage** | | | | | | | | | | |
| 27 | Heat shock protein STI  [Glycine max] | gi|41018257 | No hit | 101/72 | 14 | 9/113 | 63.9/5.81 | 65.6/6.40 | -2.43 | -1.36 |
| 37 | 31kD glycoprotein  [Glycine max] | gi|226866 | LAVEAHNIR(69)  TVNQQAFFYASER(96) | 207/40 | 12 | 12/41 | 29.8/6.72 | 25.0/6.54 | 10.64 | 9.74 |
| **Functional category 11 disease/defense** | | | | | | | | | | |
| 36 | cytosolic ascorbate peroxidase 2  [Glycine max] | gi|37196687 | GSDHLRDVFGK(45)  LAWHSAGTYDVSSK(95)  YASDEDAFFADYAEAHQK(99) | 295/41 | 30 | 21/85 | 27.2/5.65 | 27.9/5.39 | 2.86 | 1.81 |
| 41 | lectin [Glycine max] | gi|158534862 | HIGIDVNSIK(81)  VGSNGVPTSGSLGR(57)  TSYILSDTVNLK(93)  FNPVQPNIMLQK(73)  SLQTVAIEFDTFSNK(114)  ALYAAPIQIWDSETGK(96)  SLQTVAIEFDTFSNKK(94) | 608/43 | 28 | 22/82 | 30.0/5.93 | 33.4/5.90 | -1.89 | -2.02 |
| 45 | ascorbate peroxidase 2  [Glycine max] | gi|1336082 | YASDEDAFFADYAEAHQK(87) | 167/39 | 33 | 21/99 | 34.1/5.78 | 34.9/5.61 | 10.12 | 8.49 |
| 46 | ascorbate peroxidase  [Glycine max] | gi|310561 | GSDHLRDVFGK(55)  LAWHSAGTFDKGTK(61)  AMGLTDQDIVALSGGHTIGAAHK(44) | 231/41 | 30 | 23/81 | 27.1/5.51 | 30.3/4.92 | 7.51 | 7.09 |
| **Functional category 20 secondary metabolism** | | | | | | | | | | |
| 43 | myo-inositol-1-phosphate synthase  [Glycine max] | gi|84311237 | AMLENILR(46)  VVVLWTANTER(64)  VGSFQGEEIYAPFK(103)  MFIESFKVESPNVK(102)  ACVGLAPENNMILEYK(105)  DKIQQANYFGSLTQASAIR(41) | 534/43 | 19 | 45/77 | 56.6/5.46 | 57.7/5.77 | -3.10 | -2.53 |
| **Functional category 12 unknown** | | | | | | | | | | |
| 29 | unknown [Glycine max] | gi|255627339 | No hit | 44/40 | 6 | 11/41 | 25.1/4.46 | 34.2/4.65 | 2.48 | 1.40 |
| 39 | unknown [Glycine max] | gi|255626437 | FALLVEDLKVK(77)  VIIFGVPGAFTPTCSLK(83)  YTNALGLELDLTDKGLGVR(106)  VANVESGGEFTISSAEEIIK(182) | 522/42 | 54 | 22/92 | 17.5/5.41 | 17.2/5.99 | -2.64 | -1.71 |
| 40 | unknown [Glycine max] | gi|255627711 | LGLKPVTGVSR(70)  SPNSETYVIFGEAK(119)  THNGDIVGAIMELTT(111)  IEDLSSQLQTQAAQQFR(151) | 451/40 | 25 | 13/62 | 24.1/4.28 | 35.8/4.50 | -2.63 | -1.51 |
| 44 | unknown [Glycine max] | gi|255638532 | IGSVQLEPEQTWTAVQHLSIA(98) | 150/41 | 17 | 14/116 | 34.1/5.78 | 34.9/5.61 | 2.31 | 2.01 |

a) Spot No, Spot number;

b)Names and species of proteins obtained via the MASCOT software from the NCBInr database;

c) Accession No, Accession number;

d)The sequences of all the identified peptides with the corresponding ion score in brackets that were matched based on the MS/MS patterns;

e)MOWSE score probability (protein score) for the entire protein and for ions complemented by the percentage of the confidence index (C.I.);

f) SC, Sequence coverage;

g) MP/UMP indicate the number of matched and unmatched peaks for the PMF data, respectively;

h) Theor. Mr/pI shows theoretical molecular weight and pH isoelectric;

i)Exp. Mr/pI shows experimental molecular weight and isoelectric point;

j) Fold change was calculated from pathogen-challenged tissue over the control gels, which ‘-’ stands for down-regulated.
