# Supplementary material for: Proteomics study of changes in soybean lines resistant and sensitive to Phytophthora sojae
Source: Proteome Sci. 2011 Sep 7;9:52. doi: 10.1186/1477-5956-9-52 (PMC3180303; doi:10.1186/1477-5956-9-52)
Supplement: Additional file 3 — The corresponding homologues of the eleven unknown proteins. BLASTP(NCBI) was used to search the homologues of the unknowm proteins in additional file 1 and 2. The homologues with the highest homology are shown. a) The accession number of the unknown proteins in additional file 1 and 2; b) The accession number of the homologues; c) The extent to which two amino acid sequences are invariant; d) The similarities based on the scoring matrix used. [file 1477-5956-9-52-S3.DOC]

The corresponding homologues of the eleven unknown proteins

BLASTP(NCBI) was used to search the homologues of the unknown proteins in Table 1 and 2. The homologues with the highest homology are shown.

| Spot  no. | NCBI  Accession  No.^a)^ | homologue | | | | |
| --- | --- | --- | --- | --- | --- | --- |
|  |  | NCBI  Accession No.^b)^ | Protein name | organism | Idenities^c)^  % | Positives^d)^  % |
| 2 | gi|255634788 | AAO72629 | adenosine kinase-like protein | Oryza sativa Japonica Group | 82 | 90 |
| 3 | gi|255641502 | ADD51189 | globulin | Vitis berlandier×  Vitis riparia | 56 | 77 |
| 6 | gi|255628027 | NP_001149146 | soluble inorganic pyrophosphatase | Zea mays | 82 | 91 |
| 16 | gi|255634482 | XP_002877992 | indole-3-glycerol phosphate lyase IGL2 | Lamium galeobdolon | 78 | 90 |
| 21 | gi|255627339 | BAJ22388 | elongation factor 1 beta | Vigna unguiculata | 94 | 96 |
| 22 | gi|255644467 | ACY06328 | S-adenosyl-L-methionine:  caffeic acid 3-o-methyltransferase | Medicago sativa | 91 | 97 |
| 23 | gi|255641005 | CAA11075 | acid phosphatase | Glycine max | 65 | 78 |
| 29 | gi|255627339 | BAJ22388 | elongation factor 1 beta | Vigna unguiculata | 94 | 96 |
| 39 | gi|255626437 | CAQ56034 | peroxiredoxin | Pisum sativum | 93 | 99 |
| 40 | gi|255627711 | NP_190516 | NACA2(Nascent polypeptide-associated complex subunit alpha-like protein 2) | Arabidopsis thaliana | 68 | 81 |
| 44 | gi|255638532 | XP_002865061 | aldose 1-epimerase family protein | Arabidopsis lyrata subsp. Lyrata | 74 | 84 |

a) The accession number of the unknown proteins in Tables 1 and 2.

b) The accession number of the homologues.

c) The extent to which two amino acid sequences are invariant.

d) The similarities based on the scoring matrix used.
